# Enteroviruses and T1D: Is It the Virus, the Genes or Both which Cause T1D

**DOI:** 10.3390/microorganisms8071017

**Published:** 2020-07-08

**Authors:** Shirin Geravandi, Huan Liu, Kathrin Maedler

**Affiliations:** Centre for Biomolecular Interactions Bremen, University of Bremen, 28359 Bremen, Germany; geravand@uni-bremen.de (S.G.); liuhuan2012@yahoo.com (H.L.)

**Keywords:** type 1 diabetes, enterovirus, coxsackievirus, beta-cell, HLA, IF1H1, TLR3, IFIH1, YAP, Hippo

## Abstract

Type 1 diabetes (T1D) is a chronic autoimmune disorder that results from the selective destruction of insulin-producing β-cells in the pancreas. Up to now, the mechanisms triggering the initiation and progression of the disease are, in their complexity, not fully understood and imply the disruption of several tolerance networks. Viral infection is one of the environmental factors triggering diabetes, which is initially based on the observation that the disease’s incidence follows a periodic pattern within the population. Moreover, the strong correlation of genetic susceptibility is a prerequisite for enteroviral infection associated islet autoimmunity. Epidemiological data and clinical findings indicate enteroviral infections, mainly of the coxsackie B virus family, as potential pathogenic mechanisms to trigger the autoimmune reaction towards β-cells, resulting in the boost of inflammation following β-cell destruction and the onset of T1D. This review discusses previously identified virus-associated genetics and pathways of β-cell destruction. Is it the virus itself which leads to β-cell destruction and T1D progression? Or is it genetic, so that the virus may activate auto-immunity and β-cell destruction only in genetically predisposed individuals?

## 1. Introduction

*T1D (type 1 diabetes) results from a complex interplay of a multi-genetic predisposition and environmental factors.* We have read similar phrases before which are valid for numerous diseases and pathological mechanisms. Saying this is the same for diabetes as well as for any other autoimmune disease: (1) we do not really know what the real cause of the disease is and (2) apparently, there is no single cause for the disease. Thus, this phrase does describe T1D: it results from multiple triggers, which makes the disease very complex. Research has been able to identify many drivers of the disease in the past, such as the initiation of autoimmunity, paths of β-cell destruction, genetic mutations associated with the one (autoimmunity) or the other (β-cell death), or both [1,2,3,4,5].

However, we are still seeking the salient event which finally, through multiple cascades, leads to β-cell failure, loss in insulin production and secretion and, subsequently, hyperglycemia. Protection of the β-cell and prevention of diabetes before its clinical manifestation can be achieved only if the initiators are identified.

What we also know from intensive research is that T1D is a heterogeneous disease. Over the past decades, childhood T1D has increased worldwide at an estimated average annual rate of 3.9%; such doubling during the last 20 years is too high to result only from genetic causes [5,6,7]. Firstly, the concordance rate between monogenetic twins is only about 50% [8]. Secondly, epidemiological studies have shown that the disease’s incidence follows a periodic pattern within the population [5,6,9] with a significant geographical variation [6].

Support of a putative role for viral infections in the development of T1D comes from epidemiological studies, which have uncovered the seasonal pattern of disease presentation after enterovirus epidemics [6]. Specifically, enteroviruses have been made responsible as an initiator of autoimmunity as well as β-cell failure from epidemiological, pathological and in vitro studies [10,11,12].

Virus pathology *per se* commonly shows heterogeneity in its outcome, as it causes severe disease only in some affected patients. The current SARS-CoV2 pandemic in 2019/2020 is an overwhelming example of the array of outcomes of virus infection in different people, depending, e.g., on age, genetic background and pre-existing disease, from asymptomatic to pathologic [13]. There is a bidirectional relationship between Covid-19 and diabetes [14]. Firstly, several rapid communications have associated SARS-CoV2 with acute-onset diabetes [14,15], and, secondly, patients with diabetes are at greater risk for severe Covid-19 illness.

Obviously, T1D is not an acute infectious viral disease, as scenarios of massive infection in the pancreas have never been observed in T1D. The virus is lytic to β-cells in vitro, but such has not been detected in vivo, where rather a persistent infection may trigger the immune response. Most of us have had an asymptomatic enteroviral infection during childhood which did not end up causing T1D. With their positive-sense single stranded RNA genome, coxsackieviruses from the family of picornaviridae are widely spread viruses all over the world ranging from 7–22% in Greece and up to 50% and 80% in Montreal and in parts of China, respectively [16]. They most commonly cause hand-foot-and-mouth disease, producing flu-like symptoms, but also have the ability to infect the pancreas, heart and CNS. 

Together with an environmental factor, an additional factor is needed to potentiate the susceptibility to enteroviral infections to finally trigger autoimmunity and β-cell destruction, i.e., a certain genetic predisposition. Mutations have been found to either impair virus clearance upon infection, or, oppositely, to increase viral response by inducing a storm of cytokines, which will then destroy the β-cells which are vulnerable to inflammation.

## 2. Seasonal Patterns of Viral and Autoimmune Diseases

More than 60 infectious diseases have been associated with seasonal patterns, identified by a systematic search for “seasonality” from a list of communicable diseases from the Centers for Disease Control and Prevention (CDC), World Health Organization (WHO), and the European Centre for Disease Prevention and Control [17]. The flu season in the winter of the Northern Hemisphere is the most classic. As enteroviruses and especially coxsackieviruses have multiple serotypes, they cause a broad spectrum of diseases and peak at different times; however, clear seasonality has also been reported for Coxsackie B3 and B4 [17,18].

The seasonal drive is complex and multifarious. There is not only the seasonal viral exposure, but also environmental conditions such as climate (temperature, hours of daylight and sunshine) and human seasonal behavioral, i.e., diet and exercise, which reflects on the host’s immune system status and makes us more prone to infection, e.g., to flu in the winter.

In the similar way, most autoimmune diseases “go viral” seasonally, e.g., T1D, multiple sclerosis (MS), systemic lupus erythematosus (SLE), psoriasis, and rheumatoid arthritis (RA), inflammatory bowel diseases (IBD), autoimmune liver diseases (ALDs), autoimmune thyroid disease (AITD), coeliac disease, Sjögren’s syndrome (SS) and systemic sclerosis (SSc) [19]. First reported by Franklin Adams in 1926, disease breaks out in the winter season “immediately after such an infection” [9], and this has been later confirmed in large studies [20,21,22]. T1D diagnosis peaks in the colder months of late autumn to early spring, while it drops in the summer. Such seasonality disappears in regions closer to the equator. Unfortunately, sparse epidemiological data are available from equatorial regions [23], which do not allow any speculation on differences in the T1D incidence *per se*. 

**Figure 1:**
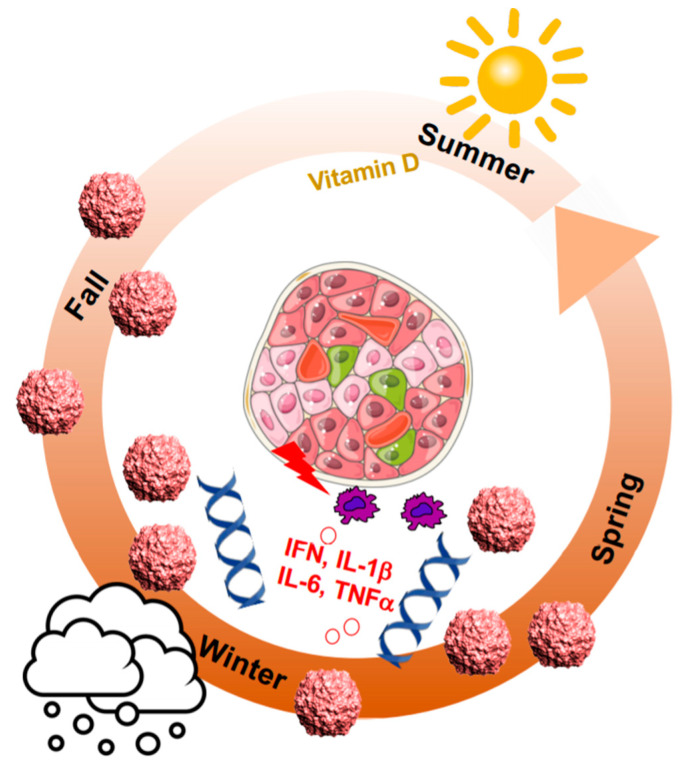
Not only environmental factors but also gene regulation show seasonal patterns. T1D (type 1 diabetes) diagnosis peaks in the colder months of late autumn to early spring, where viral infections come together with less sunlight exposure, less exercise outside, a change in diet together with an increase in pro-inflammatory cytokines and a change towards pro-inflammatory gene networks.

In the Finish DIPP cohort study, the appearance of autoantibodies showed a seasonal pattern with a significantly higher proportion in the fall and winter [24]. Thus, autoimmunity follows the same pattern as viral infection and may not just be directly caused by virus infection, but rather by a combination of unfavorable events at the same time, i.e., higher inflammation in the winter, when diet often changes to sweeter and fattier food with less exercise outside and low vitamin D levels because of limited sunlight exposure, which are all factors that have been independently shown to be associated with T1D [19] (Figure 1). Furthermore, there is the increased risk for another auto-immune disease [25]. 

Each of the single factors as a sole initiator for autoimmunity and T1D have been debated and thus, such single factor is unlikely to cause T1D. Early studies from Finland within the DiMe and DIPP cohorts have shown the association of enterovirus infection with autoimmunity and T1D [26,27,28,29], while this is not supported by previous results from the DAISY [30] and BABYDIAB [31] cohorts. Another example comes from vitamin D: while several studies show a correlation of lower levels of vitamin D with the onset of T1D [25,32], this was not confirmed by others, and several formulations of vitamin D supplementation could not reduce disease progression [33]. Crucially, it may be the seasonal change in vitamin D metabolism together with changes in the expression of its vitamin D receptor [34] that serve as the additional factors for autoimmune disease predisposition. Using large gene expression datasets from the German BABYDIET, Australia, United Kingdom/Ireland, United States and Iceland cohorts, a previous study also shows seasonal patterns in gene regulation [34]. Gene expression of both the vitamin D receptor and the anti-inflammatory circadian clock regulator transcription factor, BMAL1 (ARNTL1), is lowest in the winter [34], which promotes inflammation through increased levels of soluble IL-6 receptor and C-reactive protein [34]. Several studies in mice and isolated islets show that BMAL1 depletion impairs β-cell survival and disturbs a coordinated insulin secretion which may trigger the onset of diabetes due to defective β-cell function [35,36]. Conversely, BMAL1 is severely depleted in islets from patients with type 2 diabetes (T2D) and disrupted by IL-1β exposure of islets in vitro [37]. This suggests a direct causative role for depleted BMAL1 in inflammation and β-cell failure. Physiologically, the circadian clock would inhibit inflammation and also prevent the cell from hypoxia, as shown in the heart [38]. Thus, reduction in BMAL1 disables the cellular antioxidant response and increases HIF-1α and ROS accumulation in immune cells, which would further induce the production of proinflammatory cytokines, i.e., TNFα, IL-1β [39] from macrophages, dendritic cells as well as from β-cells themselves [40,41]. The direct cross-talk of transcription factors regulating clock genes (BMAL; ARNTL1) and hypoxia (HIF1α; ARNT) can have fatal consequences. Both belong to same family of PAS-domain, helix-loop-helix transcription factors and share some overlapping DNA binding sites [38,42]. HIF-1α mutations have not only been shown for T1D but also for many other autoimmune diseases [43] and thus again link seasonal changes with genetic predisposition for autoimmune disease. This is especially deleterious for the β-cell with its very low expression of antioxidants and high expression of cytokine and Toll-like receptors [44]. Any increased inflammation may predispose a body to β-cell failure, and thus it may not be the seasonal virus spread alone which causes auto-immunity but rather the pro-inflammatory environment in the host which potentiates β-cell failure with subsequent diabetes initiation. As such, this may only happen in genetically predisposed individuals. All three events together (viruses, the pro-inflammatory milieu in the host and the genetic profile) and their seasonality in their regulation may then initiate β-cell failure and auto-immunity.

## 3. HLA Class I and Class II Are Major Determiners for T1D

The strongest genetic risk factors for T1D are located in the major histocompatibility complex (MHC, also called the human leukocyte antigen: HLA) class II on chromosome 6, with the predisposing HLA class II haplotypes found in around 90% of patients with T1D [45]; the specific combination of HLA II alleles HLA-DRB1*03 (DR3) or HLA-DRB1*04 (DR4) with DQB1*03:02 (DQ8) confer the highest risk for T1D (for details on HLA susceptibility please see an excellent previous review [46]). 

In addition, susceptibility loci also in the HLA I region contribute to T1D [46,47,48] and their direct association with the age of T1D onset has been shown in several studies [46,47,49]. Predisposing alleles correlate with a younger age, and a protective allele with an older age at onset [47]. Children diagnosed at a very young age usually have a more severe T1D than those diagnosed as teenagers or young adults. Early T1D onset (≤5 years) can predict T1D severity, especially for diabetic complications such as retinopathy [50]. One could assume from these studies that the predisposing HLA class I alleles do not only correlate with age, but also with diabetes severity, although this has not been directly addressed in previous studies. For a possible similar correlation of HLA class II risk alleles with age of onset or severity of disease, only few study results are available. Valdes et al. reported that a DRB1-DQB1 HLA class II at risk allele contributes to the age at onset of T1D. However, a pure prediction of the disease onset from HLA alleles alone has been difficult among populations, since many more factors and their combination, i.e., T1D genetics and auto-antibodies play a major role [47].

The very early appearance of asymptomatic autoimmunity and its strong relationship with age and disease severity was found in all the large prospective T1D studies: BABYDIAB, DIPP (Diabetes Prediction and Prevention) and TEDDY (The Environmental Determinants of Diabetes in the Young). It is detected by any of the ICA, IAA, GAD, IA-2 and ZnT8 auto-antibodies and follows the exponential decay model starting in the first year of life in genetically at-risk children in affected families with first-degree relatives with T1D (FDR). Indeed, children who developed autoimmunity in the first year of life had the highest risk of T1D [51], which is further increased in those children with the high-risk HLA-DR3-DR4-DQ8 or DR4-DQ8/DR4-DQ8 genotypes [24,51].

The strong correlation of HLA-genetic susceptibility as a prerequisite for enteroviral infection-associated islet autoimmunity was depicted many years ago in the Finish DiMe study: children with a high-risk HLA allele converted to ICA positivity during enteroviral infection more often than those without HLA risk [27]. Further results from the DiMe (Childhood Diabetes in Finland) and DIPP studies show increased islet auto-antibody appearance with enterovirus infections during pregnancy and early childhood and their correlation to T1D progression [27,28,29]. 

**Figure 2:**
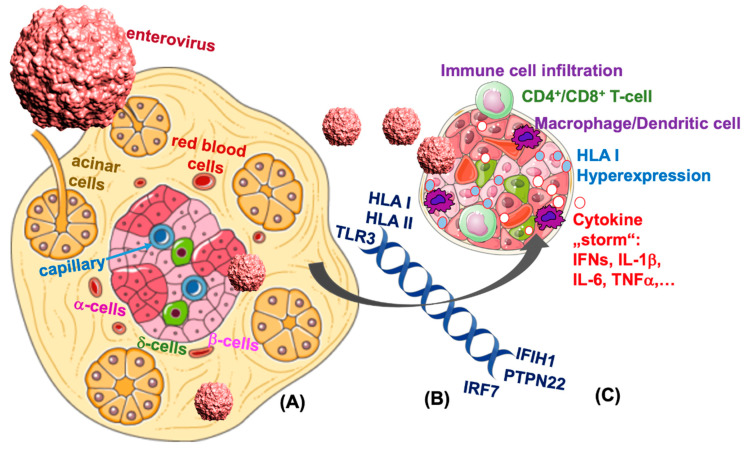
β-cell destruction in T1D is associated with viral response pathways. β-cells are highly vulnerable to enteroviral infection. (**A**) Several genetic mutations in the viral response pathway in T1D may lead to the potentiation in viral response. (**B**) A consequent “storm” of pro-inflammatory cytokines and chemokines lead to HLA I hyperexpression and attract cytotoxic T-cells and macrophages and subsequently to the loss of β-cells (**C**) and manifestation of T1D.

Confirmed in all three major T1D pancreatic tissue biobanks (EADB, Exeter Archival Diabetes Biobank; DiViD, Diabetes Virus Detection Study; and nPOD, Network for Pancreatic Organ Donors with Diabetes), the age of onset determines the number of cases with any left residual beta-cells, i.e., an older age of T1D onset strongly correlates with more remaining β-cells and children with diabetes onset < 7 years have fewer β-cells left than at the onset 7–12, and again fewer than those diagnosed at >13 years [52]. Usually seen near disease onset, i.e., within the first 7 years of diagnosis and found located and “hyperexpressed” on the surface of β-cells in T1D [53], HLA I molecules present antigens to activated cytotoxic CD8 T-cells which then lead to islet infiltration and all together to subsequent β-cell destruction (Figure 2). It is therefore possible that such HLA I hyperexpression may coincide with β-cell failure. Although the stimulus for β-cell specific HLA I hyperexpression in vivo is not clear yet, it is often associated with enteroviral infection, indirectly reported based on viral capsid protein immunofluorescence in insulin containing islet (ICI) clusters [52] as well as insulitis. Histological analyses of the human T1D pancreas show all, viral capsid VP1, IFNα, the major cytokine induced by viral infection, and HLA I expressed in or within the islet proximity [52,53]. Mechanistically shown in islets in vitro, enterovirus-induced IFNα [54] leads to β-cell upregulation of HLA class I [55,56]. IFNα-mediated HLA class I induces inflammation and ER stress, but is alone insufficient to cause beta-cell apoptosis. Additional exposure of islets to the pro-inflammatory cytokine IL-1β potentiates β-cell apoptosis [56], suggesting the necessity of a complex pro-inflammatory milieu to induce β-cell failure.

It is important to note that the association of HLA was not only identified for T1D, but for many other autoimmune diseases, i.e., rheumatoid arthritis, celiac disease and multiple sclerosis [19,57] which assumes that (i) physiological HLA is a prerequisite for a balanced immune regulation and (ii) enteroviral infections may lead to disturbance of such balance, through attraction of activated T-cells towards the virus’ homing tissue. 

Based on these large studies, islet autoimmunity in early life is indeed related to genetic factors and disease severity. The propensity of a very young child, i.e., <1 year to respond to environmental factors such as enteroviruses may thereby potentiate the risk to T1D progression.

## 4. Direct Evidence for Enteroviral RNA in the Pancreas

Epidemiological data and clinical findings show a correlation between enterovirus infection and the onset of T1D [6,58]. In 1969, Taylor’s lab reported the presence of neutralizing anti-coxsackievirus B4 antibodies in the serum of patients with T1D [59]. Since then, enterovirus infections, mainly of the coxsackie B virus (CVB) family, were hypothesized as a potential pathogenic mechanism to trigger the autoimmune reaction to β-cells, resulting in the destruction of β-cells [54,60] and the onset of T1D [61,62]. Following the isolation of CVB4 from a pancreas autopsy of a 10-year-old boy with T1D [63], many large studies tried to identify the virus directly from the T1D pancreas.

In newly diagnosed T1D patients of the DiViD study (3–9 weeks after T1D onset), VP1 was detected in biopsy pancreases in all patients in 1.7% of the islets. It is possible that such a 100% correlation of VP1 and T1D was observed because of a higher expression at diagnosis, which would decline at later stages [64], however such a hypothesis would need to be experimentally proven. Furthermore, HLA I expression was found in all patients. Viral RNA in the frozen pancreas was only found in one T1D patient and from cultured enriched islets in only 4 of 6 patients at a very low concentration (by PCR, >40 cycles), which shows no evidence of an acute but, if any, rather a low-grade infection. In confirmation with several previous studies [65], classical RT-PCR was not sensitive enough for the analysis of a viral infection, which only occurs in few cells within the whole pancreas. RNA sequencing from the whole pancreas could not identify any viral sequences, again suggesting the threshold of the presence of viral sequences compared to all other genes as sparse to be identified by classical RNASeq methods. Nevertheless, several approaches have confirmed the presence of enteroviruses both in the circulation and in islets of T1D patients [64,66,67,68,69,70], however, because of a very low expression, many attempts have failed to characterize the localization and the specific enteroviral sequences through PCR-based methods in the pancreas.

Enrichment strategies are necessary to detect such low-grade infection, e.g., amplification of viruses by preculturing human leucocytes from patients with T1D and subsequent RT-PCR analysis [71] or by the elegant viral-capture sequencing methods in which viral sequences are enriched before sequencing, that enable the identification of enteroviruses in stool samples from islet auto-antibody positive children [72].

Viruses that have a specific tropism within the islets could cause the onset of the disease not only by direct cytolysis but also by triggering the host immune response [73]. The presence of several CVB viruses, including CVB4, together with the Coxsackie-adenovirus receptor (CAR) in the β-cell, support the connection of viral infection with T1D. Coxsackieviruses induce a persistent, slowly-replicating infection; this may result from alterations to the viral genome during the progress of infection, such as naturally occurring 5′-deletions [74,75,76]. Because of several such limitations to the detection of enteroviruses, we have previously established an adapted method to target single RNA molecules with short (~20 nucleotides) fluorescently labeled oligonucleotides in situ. Probes consist of a mixture of 40 short oligonucleotides covering the whole length of the viral genome and anneal to common regions of the RNA genome of the coxsackievirus family [77]. This enables targeting single RNA molecules. Short labeled oligo RNA probes are more resistant to RNAse, and RNA detection is less affected by target RNA degradation and fragmentation. Through the availability of the well-characterized cohort of human pancreatic donor tissue established by nPOD [78], viral mRNA can be detected in the T1D pancreas with high sensitivity, specificity and accuracy and at lower viral loads than by classical immunostaining and even PCR [77,79]. Further ongoing studies of pancreas sections revealed remarkable significance of viral RNA expression in T1D pancreata, compared to controls without T1D [80].

**Figure 3:**
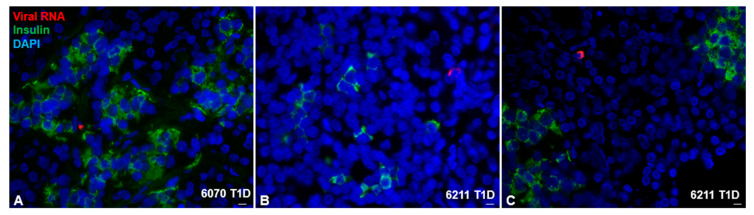
Coxsackieviral RNA in the T1D pancreas. Representative images of T1D donors 6070 and 6211 from the nPOD cohort. Viral RNA was found within the endocrine area (**A**) and outside the islets (**B**,**C**) shown by co-staining of viral RNA probes (red), insulin (green) and DAPI (nuclei; blue). Tissues were first probed for viral RNA, and then stained for insulin after a previously established protocol ([77]; Busse et al.). Scale bar depicts 10 µm.

Using this method, we have analyzed whole pancreas sections and quantified enteroviral mRNA by unbiased scans and identified viral mRNA distributed not specifically within or in proximity to islets; enteroviral mRNA was evident through individual dots in single cells throughout the pancreas (Figure 3). Such observation is in contrast to VP1 immunohistochemistry in the pancreas [81], which mostly detected VP1 positivity in or near islets. Famously referred to as the “streetlight effect” [82], it is difficult to find what we search for in the dark, and thus, it is possible that several antibody-based stainings were preferentially observed in islets, although the staining has been carefully re-evaluated and VP1 correlates with hyperexpression of HLA class I in islets [83]. The commonly used DAKO-VP1-Ab detects several other antigens in addition to VP1 and/or exocrine enzymes may degrade enteroviral proteins and thus prevent their detection in the pancreas [84]. 

Early studies, where C57BL/6 mice were infected with CVB3, also observed viral infection localization in the pancreas in the acinar cells, together with severe inflammation and acinar cell destruction [85]. Despite the well-known differences in enteroviruses’ tropism in the pancreas in mice and humans [85], such observation is in line with the decreased acinar cell number and acinar tissue mass reported in numerous studies from human T1D pancreases [86,87,88].

**Figure 4:**
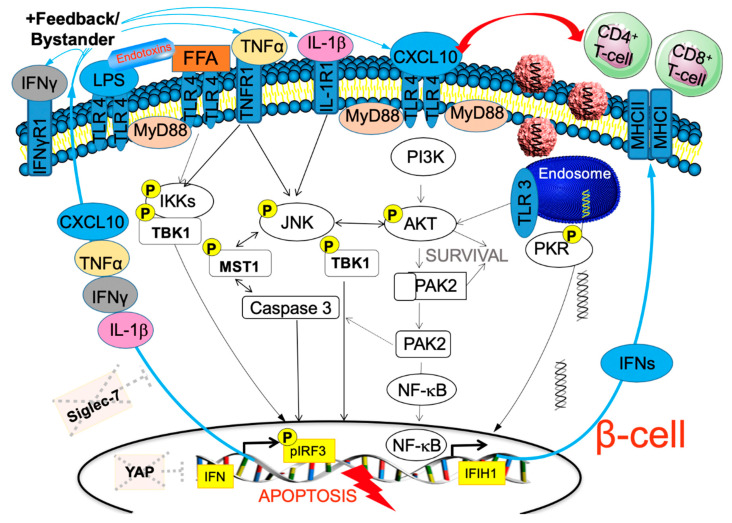
β-cell in the storm. Our hypothetical model on how chronic potentiation of proinflammatory pathways leads to β-cell destruction. Coxsackieviruses enter the β-cell through the Coxsackie–adenovirus receptor (CAR) and bind to endosomal TLR3. While the virus promotes the AKT-JNK axis for initial host cell survival, parallel activation of viral response pathways through PKR-TBK-IRF3 leads to the transcriptional activation of the IFN response and production of interferons, which increase surface MHCs, recognized by cytotoxic CD8- and CD4-T-cells causing “bystander damage”, and β-cell apoptosis through a “storm” of cytokines and chemokines, which all find their receptors on the surface of the β-cell, and a vicious cycle is initiated with the full activation of the apoptotic machinery including JNK-MST1-Caspase 3-NFκB. Bacterial toxins as well as chronically elevated free fatty acids (FFA) are also associated with β-cell damage and act through TLR4 activation and similar downstream pro-inflammatory pathways. While many cells can counteract such damage cycles with a potent survival machinery, the β-cell is deficient of the Hippo terminator YAP, which would balance the viral IRF3 response. Furthermore, Siglec-7, which balances immune activation, is diminished in a chronic diabetogenic pro-inflammatory milieu in the β-cell.

Rather than from the virus itself, β-cell destruction may result from “bystander” damage [89,90], where coxsackie virus infection may lead to a storm of inflammation in cells like the β-cell, which carry an enormous amount of pattern recognition (such as TLR3 and TLR4), cytokine (such as IL-1R1), and chemokine receptors on their surface [44]. Their activation by viruses and by cellular viral responses stop viral replication on one hand, but induce tissue damage on the other. In addition, interferons accelerate expression of surface HLA-I molecules and thus activation of auto-reactive T-cells against β-cells (Figure 2 and Figure 4). T-cell activation through non-T-cell receptors (“bystander damage”) [89] is limited to viral infection [91], where β-cell apoptosis is triggered by viral response products, e.g., cytokines and chemokines [92]. Such a pro-inflammatory environment has also been shown to alter the composition of the islet extracellular matrix, which may further facilitate T-cell migration towards pancreatic islets [93]. The specific and severe β-cell destruction then occurs through their special vulnerability towards an array of cytokines and chemokines such as interleukin (IL)-1β, interferon (IFN)-γ, tumor necrosis factor (TNF)-α and CXCL10 [94], which induce β-cell destruction in response to viral infection in human islets [60,95] (Figure 4). It is also possible that multiple infections during childhood each time contribute to potentiating the immune response and then lead to β-cell destruction, autoimmunity and T1D. 

## 5. Enteroviral Infection and T1D: Results from the TEDDY Study

Recent results from the large multi-center TEDDY cohort study provided important confirmation of the association of enteroviral infection and islet autoimmunity [96]. Direct next-generation sequencing of stool samples as well as analyses subsequent to cell culture amplification of enteroviruses identified an array of DNA and RNA viruses.

The study confirmed that enterovirus B infections (EVB) were associated with islet autoimmunity, but also examined the role of length of infection since sequential stools from children were available. An association with islet autoimmunity was detected with long-duration enterovirus B infections, indicated by prolonged shedding of the same virus in multiple stool samples. In contrast, multiple independent short-term enterovirus B infections without prolonged shedding neither correlated with autoimmunity nor with T1D progression.

The results of this study indeed reproduce a correlation of enteroviral infection and autoimmunity: that the duration of the virus load detectable in stool samples determines the progression to autoimmunity. Mechanistically, one can assume from this and many previous studies that the virus may trigger autoimmunity, but is not conclusively linked to further T1D progression.

This is in line with data showing that the enteroviral signaling cascade, which leads to the IFN response, is increased before auto-antibody conversion and T1D (see above) [97], which again suggests virus infection and the boosted IFN response as primary event toward autoimmunity. It is very likely that the longer duration of enterovirus abidance in the host is defined by the genes and their unfavorable seasonal changes.

Once a host is found, the virus creates a variety of smart mechanisms to escape from anti-viral immune response through persistent infection, e.g., blocking autophagy in order to remain in the cell [16]. Dysfunctional autophagy as a feature of both T1D and T2D [98] supports such hypothesis. Enteroviral B’s typical 5′ terminal genomic deletions observed in cardiomyocytes [99] and in the pancreas [76] may lead to a long term stay of viruses in the cell without causing lysis. This probably enables detection of viral RNA in autopsy pancreata even a long time after occurrence of islet auto-antibodies [80] as well as after T1D diagnosis [77] in morphologically normal appearing cells.

Highly sensitive virus-captured sequencing methods from stool samples also confirm the association of enteroviral infection with islet autoimmunity [72], and enteroviral amplification-enrichment cultures of leukocytes and of cells from duodenal biopsies showed the correlation of enteroviruses B and T1D. A significant association between enterovirus and subsequent risk of autoimmunity in celiac disease was also found in TEDDY and other previous studies [57,100], where enteroviral positive stool samples correlated with celiac disease only after introduction of gluten to the babies’ diet [57], and higher amounts of gluten consumption potentiated the effect of enteroviruses on the risk of coeliac disease autoimmunity [100], indicating the necessity of the initial autoimmunity trigger.

## 6. TLR3 Signaling Leads to Enterovirus-Induced β-Cell Destruction

The innate immune response to virus infection initiates as a fingerprint with the sensing of viral pathogen-associated molecular patterns (PAMP). Such recognition is mediated by the activation of host’s pattern recognition receptors (PRR) such as Toll-like receptors (TLR) on the surface of cellular membranes and cytosolic receptors including RIG-like receptors (RLR), nucleotide-binding domain-leucine-rich repeat-containing molecules (NLR) and RNA-activated protein kinase R (PKR) [101]. Many studies show that the onset of diabetes is triggered through PRRs [102,103,104], and PRRs have been identified as susceptibility factors for diabetes progression in genetic studies [105,106,107]. Most of the today’s described 10 human TLRs, namely TLR2-4 and 6-9 have been associated with T1D or/and T2D [108,109]. There is a strong correlation of the most TLR3 polymorphisms with T1D in several [107,110] but not in all studies [111].

TLRs are used by the immune system for pathogen clearance. The endosomal receptor TLR3, found not only in immune but also various non-immune cells such as the β-cell, is one of the signaling complexes implicated in viral-mediated β-cell death, is highly expressed in the pancreas of patients with T1D [112] and is found enhanced in human islets by IFN exposure [113]. Once viral RNA is recognized by TLR3, the TLR3-TANK binding kinase 1 (TBK1)-IFN-regulatory factor (IRF)3/7 signaling axis is activated [114]; the virus initially induces AKT [60] to make sure that its host survives but later cross-talks with JNK result in activation and translocation of NF-κB subunits to the nucleus (Figure 4).

Downstream of the viral response pathway is the C-X-C motif chemokine 10 (CXCL10) which promotes human β-cell apoptosis [94]. CXCL10 is localized in infected islets [115] in both canonical and fulminant T1D early in disease progression [44,94] and thus is suggested as a clinical marker for diabetes onset [116]. The cascade finally ends in the secretion of proinflammatory chemokines and cytokines, which further potentiate inflammation and β-cell apoptosis pathways (Figure 4).

Several studies in mice have shown that TLR3 is an essential element of T1D development in response to viral infection. As a detector of viral signatures, TLR3 is needed for the anti-viral response, and, naturally, will promote cytokine signaling. These two apparent conflicting effects towards beta-cell survival may provide reasons for various different results in mice and imply that a highly balanced physiological function of viral sensors is necessary to prevent damage to β-cells. TLR3 signals contribute to the host’s survival, as CVB4 [117] or encephalomyocarditis virus [118] infections are highly mortal to TLR3 knockout mice due to the impaired antiviral response machinery. Although they present a reduced pro-inflammatory milieu, surviving mice develop T1D [117]. Other studies show that TLR3 knockout in NOD mice has no effect on the incidence of diabetes at a basal level [119] and that CVB4-infected TLR3 knockout NOD mice show lower diabetes incidence [120]. In the absence of TRIF, a prominent downstream protein in the TLR3 cascade, mice are also protected from the development of T1D by changing the gut microbiota [121].

In summary, pattern recognition receptors identify viral antigens to trigger the host defense. TLR3 signaling through multiple loops leads to virus-mediated inflammatory response, and ongoing inflammation further potentiates the cytokine response through multiple cytokine and chemokine receptors expressed in the β-cell, and finally to β-cell apoptosis in vitro. However, as many examples show, mutations in a single PRR, e.g., TLR3, or its activation alone will not ultimately cause T1D, but may rather act within a pro-inflammatory network to potentiate T1D progression (Figure 4). A future research target towards prevention could therefore be specific miRNAs, as many of them which are differentially expressed in T1D patients [122] are involved in the regulation of the innate as well as the adaptive immunity through TLR signaling [123].

## 7. IFN-Inducible Genes Link Autoimmunity, Viral Response and β-Cell Failure in T1D

T1D is associated with over 60 genetic risk regions across the human genome, identified by genome-wide association studies (GWAS) [124], and these T1D-linked SNPs alter the expression of over 200 genes [125] involved in β-cell inflammation, function and destruction, immune activation and signaling, including viral response, Toll-like receptor, cytokines and NF-𝜅B signaling. Among them, several risks as well as protective single nucleotide polymorphisms within the interferon-induced helicase-1 (*IFIH1*) gene, which encodes the melanoma differentiation associated protein 5 (MDA5), have been identified in large studies [106,126]. IFIH1 is a cytosolic sensor of single strand viral RNA from the picornavirus family. It facilitates the interferon (IFN) response and activates the immune cells towards viral response downstream of TLR signaling. Importantly, expression of the IFN signature genes as well as the type 1 IFN response is increased in children before the T1D-associated auto-antibody conversion [97,127], which suggests a primary role of IFN signals in the activation of autoimmunity and the potentiation of β-cell destruction. In β-cells, IFN signaling leads to HLA class I hyperexpression, which is a well-studied path for T1D initiation [106,128]. *IFIH1* is ultimately associated with signals from enteroviruses; its mRNA expression is increased by CVB3 and CVB4 infection in human islets [114] and by synthetic double-stranded RNA Poly(I:C) in INS-1E β-cells [129], while *IFIH1* silencing potently lowers the chemokine response in β-cells [129]. Foremost, a diabetes-associated *IFIH1* polymorphism upregulates the IFN signature in human pancreatic islets in response to Coxsackievirus infection [130].

The upregulation of IFN-inducible genes, including *IFIH1* in genetically predisposed children, was also associated with previous upper respiratory tract infections and with increased monocytic expression of the sialic-acid binding immunoglobulin-like-lectin Siglec-1 [97]. Through the recognition of specific glycans on the cell surface, Siglecs promote cellular interactions within the immune system and with sialylated pathogens; they are important regulators of the innate and adaptive immune systems and serve as checkpoints for immune regulation and autoimmunity [131]. Through their immunoreceptor tyrosine-based inhibitory motifs (ITIMs), Siglecs balance the immune response [132]. Several members of the Siglec family do not only play a role in immune–cell–pathogen interactions, but also on the level of the β-cells regulate the inflammatory response. Siglec-7 is down-regulated in both β-cells in the pancreas from patients with T1D and T2D as well as in activated immune cells. Overexpression of Siglec-7 in diabetic islets balances the immune response by reducing cytokine production and monocyte migration, which both facilitate β-cell survival and function [133]. The evolving field of Siglecs provides a further target to modulate the excess inflammatory/IFN response as a major facilitator for autoimmunity and β-cell failure.

## 8. Why the Beta-Cell? Absence of the HIPPO Effector YAP to Balance Viral Response

Despite certain viral tropisms, viral receptors are distributed in many cells in all organs and IFN-induced viral defense mechanisms are in place, which (i) hinder viral reproduction and (ii) attract cytotoxic T-cells. In the largely non-replicative β-cells, such an increase in the IFN response seems deleterious. The intracellular antiviral defence is initiated by TBK1-IRF3-mediated interferon production (see Section 6 and Section 7 above) [134] and controlled by the Hippo terminators and transcriptional regulators YAP and TAZ [135,136,137], which negatively regulate and thus balance the antiviral immune response. Recent studies have linked YAP/TAZ with antiviral sensing [135,136,137]. YAP/TAZ associate with both TBK1 and the inhibitor of nuclear factor kappa-B kinase (IKKε), thereby blocking their activation and subsequently inhibiting IRF3-stimulated transcription of viral response genes. Thus, YAP/TAZ, besides their well-known function in the regulation of cellular contact, development, growth and proliferation as effectors of the Hippo pathway [138], can regulate the host’s cellular response. In the absence of this YAP regulation, virus sensing would trigger an extremely high and uncoordinated cytokine response, as happens in T1D, where virus-infected β-cells show highly increased cytokine production resulting in a vicious cycle and bystander damage of β-cells through their cytokine receptors (Figure 4).

One underlying reason could be the absence of YAP in adult β-cells. During endocrine cell differentiation, YAP is suppressed as soon as Ngn3 is expressed [139,140]. The lack of YAP expression correlates with the extremely low rate of β-cell proliferation and β-cell quiescence after birth and their limited regenerative capability [141]. The Hippo element YAP is sufficient to wake β-cells up from quiescence; re-expression of constitutively active YAP leads to a robust induction of human β-cell proliferation [140,142]. Similarly, TAZ is extremely low but detectable in both adult human and mouse β- and α-cells [143,144]. Bioinformatic analysis identified YAP as a selectively repressed (“disallowed”) gene in the pancreatic islet [145]; it is more repressed in purified mouse β-cells compared to α-cells [146]. Now, we hypothesize this as the reason not only for the much lower proliferative capacity of β-cells compared to any other endocrine cell type, but also for the extreme and suicidal viral response. In contrast, the Hippo kinase MST1 represses antiviral signaling and acts as negative regulator of the antiviral defense by its direct interaction and phosphorylation of IRF3 and inhibition of TBK1 [137]; however, underlying mechanisms as well as consequences on host survival are not known. Previous data from our and other labs show that Hippo is an important regulator of β-cell function and survival [139,140,147], and therefore it may also be involved in the pathological viral response associated with T1D.

## 9. Conclusions

There is large evidence for enteroviral infection initiating the auto-immune response and subsequent β-cell destruction in genetically predisposed individuals, where a viral response is boosted. As an especially vulnerable cell to inflammatory destruction and apoptosis, autoimmunity is directed to the β-cell, causing T1D. Although enteroviruses selectively and severely destroy β-cells in vitro, they are just one stimulating factor in the huge complexity of T1D, and thus, without an unphysiological genetic predisposition towards immune activation and β-cells’ inability for compensation, they would probably not cause T1D. Therefore, it is possible that enteroviral vaccination and antiviral therapies for T1D [148], although they would take away the stimulus, may alone not be sufficient to cure the disease and require combination with further β-cell protection efficacy. This is reminiscent of gluco- and lipotoxicity-mediated β-cell failure associated with T2D [149]. Although highly toxic for the β-cell in vitro, elevated glucose and free fatty acids only induce some alterations and systemic compensation as long-term consequences of obesity in vivo. However, in genetically predisposed individuals, they finally lead to T2D [150]. Similarly, neither viral infections alone nor predisposing genetic polymorphisms alone ultimately lead to T1D. As there is no single cause for T1D, we will probably not be able to successfully cure diabetes with a single drug. Rather, forces need to join for testing the efficacy of combination therapies, for example antiviral strategies [148] together with the prevention of T-cell action [151], anti-inflammation [152] and/or beta-cell protection [153].
